# A Novel Approach towards Synthesis and Characterization of Non-Cytotoxic Gold Nanoparticles Using Taurine as Capping Agent

**DOI:** 10.3390/nano10010045

**Published:** 2019-12-24

**Authors:** Akash Kumar, Nabojit Das, Neeraj Kumar Satija, Kapil Mandrah, Somendu Kumar Roy, Raja Gopal Rayavarapu

**Affiliations:** 1 Nanomaterial Toxicology Laboratory, Nanomaterial Toxicology Group, CSIR-Indian Institute of Toxicology Research (CSIR-IITR), Vishvigyan Bhawan, 31 Mahatma Gandhi Marg, Lucknow 226001, India; 2 Academy of Scientific and Innovative Research (AcSIR), Ghaziabad 201002, India; 3 Developmental Toxicology Laboratory, Systems Toxicology & Health Risk Assessment Group, CSIR-Indian Institute of Toxicology Research (CSIR-IITR), Vishvigyan Bhawan, 31 Mahatma Gandhi Marg, Lucknow 226001, India; 4 Analytical Chemistry Laboratory, Regulatory Toxicology Group, CSIR-Indian Institute of Toxicology Research (CSIR-IITR), Vishvigyan Bhawan, 31 Mahatma Gandhi Marg, Lucknow 226001, India

**Keywords:** gold nanoparticles, toxicity, capping agent, synthesis, taurine, characterization

## Abstract

Metal gold nanoparticles are of great interest due to their unique physico-chemical properties and their potential to be used as nano-probes in biosensors, drug delivery, and therapeutic applications. Currently, many capping agents are used for metal gold nanoparticles, such as cetyltrimethylammonium bromide (CTAB) and tri-sodium citrate that have been reported to be toxic and hinders biological applications. To address this issue, we report, for the first time, the use of taurine as a stable non-cytotoxic capping agent for synthesizing gold nanoparticles by using an in situ wet-chemical method. This facile method resulted in monodisperse gold nanospheres with a high yield and stability. Monodisperse gold nanospheres with average diameters of 6.9 nm and 46 nm were synthesized at a high yield with controlled morphology. Temperature played a critical role in determining the size of the taurine-capped gold nanoparticles. The subtle changes in the reaction parameters had a tremendous effect on the final size of nanoparticles and their stability. The synthesized nanoparticles were characterized by using optical spectroscopy, a ZetaSizer, a NanoSight, Fourier Transform Infrared (FTIR) spectroscopy, X-ray Diffraction (XRD), X-ray Photon Spectroscopy (XPS) and Electron Microscopy to understand their physico-chemical properties. Taurine was explored as a capping and stabilizing agent for gold nanospheres, which were evaluated for their toxicity responses towards human liver carcinoma cells (HepG2) via MTT assay.

## 1. Introduction

Plasmonic metal (noble) nanoparticles are of great interest due to their unique physico-chemical properties in the sub-100 nm size regime [1]. Metal gold nanoparticles (AuNPs) exhibit enhanced optical properties due to their intrinsic phenomena of localized surface plasmon resonance (LSPR) [2]. This phenomenon of surface plasmon resonance enables metal nanoparticles to have strong absorption and scattering properties. The light absorbed by the metal nanoparticles is converted to heat that can be used for various nanophotonic applications [3]. This feature is prominent in noble metal nanoparticles that have the surface plasmon resonance (SPR) effect which is absent in non-metallic nanoparticles. The mean free path of a nanoparticle is dependent on size and shape thus making it a critical factor for determining the physical, chemical, optical, magnetic, electronic and catalytic properties of a nanoparticle [4]. Gold nanoparticles have been extensively used in the past decade for biomedical applications such as surface-enhanced Raman spectroscopy (SERS), optical sensors, fluorescence, and sensor chips [5,6]. These technologies are based on the principle of elastic scattering properties and on the plasmon shift of metal nanoparticles [6]. Gold nanoparticles, in particular, are utilized for bioimaging, nanovehicles, and as molecular contrast agents that are suitable for the specific delivery of ligands/drugs to the desired target site. In lieu of subjection to numerous applications in biomedicine, gold nanoparticles have yet to be established for biological applications due to their potential toxicity concerns. The toxicity of gold nanoparticles is dependent on their size, shape and surface chemistry [7]. The control of chemical entities on the surface of nanoparticles in the nanoscale regime has been a challenge for translational applications. The nanoparticle stability can be enhanced by proper capping agents that provide stability and flexibility for binding affinity molecules. The nature of the capping agent determines the uptake of nanoparticles into cells, depending on their physico-chemical properties. Nobel metal nanoparticles (gold/silver/platinum) have been capped with surfactants, and thiolated polymers that have applications in biomedical research [2,8].

Despite the numerous advantages of metal nanoparticles, there are concerns regarding their safety on biological cells as reported in in-vitro/in-vivo toxicity studies [9]. In bulk form, gold is inert/non-toxic, but the manner in which these nanoparticles are developed using different synthetic procedures and the nature of capping/stabilizing agents may cause toxicity [10]. The nature of the nanoparticle depends on the synthetic approach, and several groups have previously reported on colloidal metal nanoparticle synthesis using green chemistry, photochemical, wet chemical and electrochemical methods [11,12,13]. Plant extracts have been used for synthesizing metal nanoparticles, but, unlike the procedure we used for taurine-capped metal nanoparticles, the synthetic procedure is laborious. As mentioned previously, green synthesis is mainly focused on the synthesis of nanoparticles, which has the potential to replace the existing use of hazardous reagents that are used during chemical synthesis. However, its environmental advantage alone may not be sufficient to guarantee green synthesis as a feasible solution for industrial realizations. Major obstacles in green chemical synthesis include their scalability, the removal of by-products, and purification. Till date, nanoparticles synthesized via wet-chemical approach are superior in terms of shape control and monodispersity compared to other synthetic procedures [14]. Though nanoparticles synthesized via green chemical methods through the use of plant extracts, polysaccharides, biomass, phytochemicals, and synthetic molecules are less toxic, their major disadvantages are their polydispersity, yield, purity, and metabolite formation. Most of the available commercial/laboratory scale synthesized gold nanoparticles have either trisodium citrate or surfactant as capping agents. However, in the literature it has been reported that citrate-capped gold nanospheres are not stable, tending to aggregate at a higher pH or in different ionic strength solvents/buffers [15]. Depending on the capping of nanoparticles, both cationic and anionic functional groups have been reported to be toxic to liver cancer cells at the exposed concentrations [16,17]. Cetyltrimethylammonium bromide (CTAB), a commonly used structure-directing agent for the synthesis of anisotropic/isotropic nanoparticles, is highly toxic to liver cancer cells and hinders biological applications [17]. Therefore, there are lacunae in the research regarding the selection appropriate/suitable capping agents for nanoparticles that can stabilize and provide functionality to nanoparticles for applications in nanomedicine. To address this, we report synthesis using an unexplored non-protein amino acid (taurine) that is naturally synthesized in the body by various tissues [18]. Recent studies showed the potential of bulk taurine for various biological applications [19,20,21] indicating their advantages that have promising potential for their use in nanomedicine. Additionally, it has been reported that taurine reduces hepato-toxicity and decreases reactive oxygen species (ROS) formation, unlike citrate or any other capping agents [22]. A recent report showed that taurine interacts with the noble metal silver and studied the pair’s binding capabilities [23]. However, the role of taurine in synthesizing noble metal nanoparticles via in situ wet-chemical method has never been explored in a way that can enhance these particles’ use for nanomedicine. Previous reports have shown that taurine can interact with metals and also act as cyto-protective agent in alleviating the symptoms of various pathological conditions related to cardiovascular, skeletal muscle and defective metabolism disorders [24,25]. A recent clinical trial showed reduced psychotic symptoms after administering four grams of taurine within 12 weeks in 121 young patients [25]. These beneficial uses of taurine will provide unique advantages to aid nanoparticles and will be further developed for enhanced bioavailability to address various pathological conditions.

To the best of our knowledge, this is the first report on synthesizing metal nanoparticles while using taurine as a capping agent via an in situ wet-chemical reduction method. Taurine is known to have interactions with metal ions, but its role as a capping/stabilizing agent for nanoparticles has not been reported. The bioavailability of gold nanospheres could be increased when using taurine as a capping agent, thus making it an alternative to replace cytotoxic capping agents. This novel synthesis could benefit the area of nanomedicine by aiding in the development of non-cytotoxic metal nanoparticles.

## 2. Materials and Methods

Taurine (2-aminoethanesulfonic acid), hydrogen tetrachloroaurate trihydrate (HAuCl_4_·3H_2_O), and sodium borohydride (NaBH_4_) were purchased from Tokyo Chemical Industries (TCI) TCI, Finar, and Sigma-Aldrich, respectively. All the chemicals were used as received. The liver carcinoma cell line (HepG2) from American Type Cell Culture, Virginia (USA), Dulbecco’s Modified Eagle Medium (DMEM), trypsin, phosphate buffered saline (PBS), fetal bovine serum (FBS), and antibiotics were purchased from Sigma-Aldrich. All the stock solutions were prepared with the use of double distilled water. Aqua regia was used to clean the glassware, which was further rinsed with Milli Q before using them for experimental procedures.

### 2.1. Methodology

#### 2.1.1. Optical Spectroscopy of Taurine-Capped Gold Nanospheres

The synthesized gold nanospheres were measured for plasmon bands by using optical spectroscopy. The as-prepared gold nanospheres were measured by taking 1 mL of the sample in a polystyrene cuvette. The measurements were done by using a UV-VIS-NIR spectrophotometer (Spectramax M5, Molecular Devices, San Jose, CA, USA). The gold nanospheres were measured in the wavelength range of 400–900 nm.

#### 2.1.2. Hydrodynamic Radius and Surface Charge Measurements Using Nano ZetaSizer

The hydrodynamic radius and surface charge of the metal nanoparticles were measured with a ZetaSizer (Nano ZS, Malvern, UK). One milliliter of sample was taken and sonicated for 30 s (under tapping mode) and was taken in a cuvette. The samples were sonicated and further diluted before the measurements by using Milli Q. A 1 mL syringe was used in order to disperse the solution into the cuvette, and care was taken such that no bubbles were formed in the solution. The pH of the nanoparticle samples were 3.30 and 3.00 for samples exhibiting absorbance of λ_max_ (515 nm) and λ_max_ (545 nm), respectively. Separate cuvettes (DTS1072 and DTS0012) were used to measure the surface charge and average size of nanoparticles, respectively. The absorption coefficient and refractive index of gold were prefilled in software with values of 1.520 and 0.100 respectively. All the measurements were performed at 25 °C.

#### 2.1.3. Nanoparticle Tracking Analysis (NTA) Using NanoSight

Nanoparticle tracking analysis (NTA) was used to determine the hydrodynamic radius and concentration of the nanoparticles (particles per mL), aided by visualizing the Brownian motion of nanoparticles via a camera. The nanoparticles were maintained at room temperature, and the pH was between 3.0 and 3.3 for the samples of nanoparticles. The nanoparticle sample with a dilution factor of 10^−1^ was used for analysis. A blue laser (488 nm) was used for all the measurements.

#### 2.1.4. Transmission Electron Microscopy

Taurine-capped gold nanospheres with absorbance λ_max_ values of 515 nm and 545 nm were centrifuged at 16,000× *g* and 7000× *g* for 30 min, respectively. The supernatant was discarded, and the pellet was dispersed into Milli Q. The nanoparticles were sonicated, and the diluted sample (5 μL) was drop-casted on the formvar-coated carbon grid and air dried for 2 h. The TEM imaging was done with Tecnai (FEI, The Netherlands).

#### 2.1.5. Atomic Absorption Spectroscopy (AAS)

To determine the gold metal content, a working standard of Au was prepared with a concentration of 50 mg/L by using the National Institute of Standards and Technology NIST standard of 1000 mg/L. A linear curve was plotted against serial dilutions of 1, 2, 5 and 10 mg/L. All dilutions were made by using 1% HNO_3_. For sample preparation, 100 µL of the AuNPs suspension was diluted with 1% HNO_3_, making the volume up to 10 mL. Analysis was performed on an atomic absorption spectrophotometer (ZEEnit 700, Analytik Jena AG, Germany) equipped with a high intensity hollow cathode lamp of Au, and manufacturer brand software (Win AAS) was used for data integration and the resultant outcome. A 1 mL sample solution was sprayed over a high temperature flame for atomization; the metal (Au) content was subsequently estimated with a linear equation.

#### 2.1.6. FTIR and Raman Spectroscopy

The solutions of nanoparticles were centrifuged and further lyophilized to obtain nanopowders. The nanopowders were characterized by using FTIR (Thermo Scientific, Nicolet iS5) and Raman spectroscopy (Renishaw In Via). Five milligrams of nanopowder were placed on the holder that was clean and dried for the FTIR measurements. Raman measurements were done by depositing 10 mg of nanopowder onto a dry holder that was purged with inert gas. An excitation wavelength of 514 nm was used to understand the molecular vibrations between taurine and the gold metal surface.

#### 2.1.7. XRD and XPS Analysis

Thirty milligrams (powder form) of taurine capped gold nanoparticles were placed onto the holder to measure the crystal structure and size using PANalytical XRD.

To determine the surface chemistry, XPS (PHI 5000 Versa Probe II, FEI Inc) was used to validate the interaction and binding energy between taurine and gold metal surface.

### 2.2. Cell Culture

The hepatocellular carcinoma (HepG2) cell line was obtained from American Type Cell Culture Collection (ATCC, Virginia, USA). The cells were maintained in DMEM supplemented with 10% (*v*/*v*) FBS and 1% Pen–Strep as an antibiotic to prevent bacterial contamination. The cells were cultured at 37 °C with 5% CO_2_ in a 90% humidified incubator.

#### Cell Viability Assay

To evaluate the safety of the taurine-capped gold nanoparticles, we tested cell viability by using 3-(4,5-Dimethylthiazol-2-yl)-2,5-diphenyltetrazolium bromide (MTT) assay [26] on liver carcinoma cell line, as 1 × 10^4^ cells were seeded in 96 well-plate and incubated overnight at 37 °C in a CO_2_ incubator. The counting of cells was carried out by using a hemocytometer. The control group only consisted of cells that were not treated with nanoparticles. The concentration (particles/mL) of the taurine-capped metal nanoparticles was calculated as per a previously reported method [27]. The samples of the taurine-capped metal gold nanospheres were diluted to different concentrations (1 × 10^11^–1 × 10^8^ particles/mL) in media. The cells were exposed for 24 h with nanoparticles. After 24 h of exposure, 10 μL of MTT solution (5 mg/mL) were added to each well and left to incubate at 37 °C in 5% CO_2_ for 2 h. After the purple color precipitate was visible, 100 μL of acidic sodium dodecyl sulfate (SDS) (10% SDS + 0.01N HCl) was added to each well and left overnight at 37 °C. Absorbance was measured at 570 nm and 690 nm (test and reference wavelength, respectively) using spectrophotometer (Spectra Max M5, Molecular Devices, San Jose, CA, USA). The assay was carried out in quadruplicate.

### 2.3. Nanoparticle Synthesis

**Figure 1:**
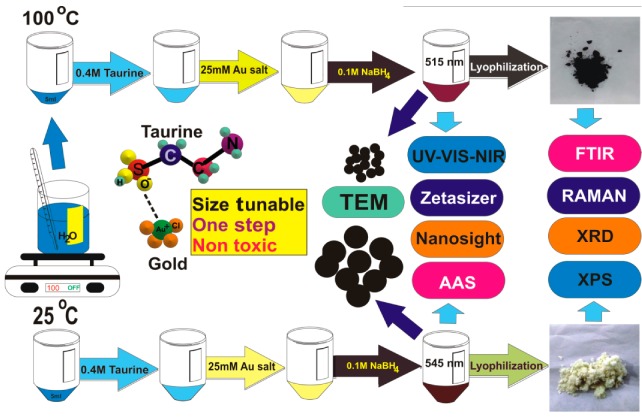
Schematic diagram of the taurine-capped gold nanospheres that were synthesized with a wet-chemical method in a one-step/one-pot method. The reaction parameters controlled the morphology and size of the taurine-capped gold nanospheres, both of which were dependent on temperature. The absorption bands of the different sizes of gold nanospheres were 515 nm and 545 nm, and these were dependent on their size. Detailed characterizations of the taurine-capped gold nanospheres were done by using various standard techniques to determine their absorbance, size, shape, charge, surface composition, binding energy and crystalline structure. The taurine-capped gold nanospheres were obtained both in solution and nanopowder form with a high yield and monodispersity.

The taurine-capped metal gold nanospheres were synthesized by using the wet chemical reduction method. The experiment was carried out at different temperatures and was a critical factor in determining the size of gold nanospheres. The schematic representation of the synthesis protocol is shown in Figure 1. In a typical reaction, 5 mL of distilled water was separately transferred to two tubes (one at boiling temperature and the other at room temperature). Consequently, 2 mL of 0.4 M taurine was added, followed by the addition of 500 µL of 0.025 M chloroauric acid in both the tubes. Finally, 1 mL and 0.5 mL of 0.1 M NaBH_4_ was added to the solutions. The color changed from yellow to reddish-brown after the addition of NaBH_4_ due to the reduction of Au^3+^ to Au^0^. Our aim was to develop non-cytotoxic metal nanoparticles by using a non-toxic or less toxic molecule that could act as a capping agent. To achieve this, we synthesized metal gold nanospheres without taurine (referred as control), which resulted in unstable nanoparticles with a lesser shelf-life that aggregated within few days. However, in presence of taurine, the metal gold nanospheres were stable for more than a month, and their shelf-life was better even at different temperatures. The gold nanoparticles were centrifuged twice to remove excess sodium borohydride, which was used as a strong reducing agent. The purification prevented the formation of the oxidized by-products of NaBH_4_, thus leaving only the taurine-capped gold nanoparticles in the colloidal solution. The nanoparticle pellets obtained after centrifugation were lyophilized, thus resulting in nanopowders. These nanopowders were further characterized by using FTIR, XRD, Raman and XPS techniques to determine their physico-chemical properties.

## 3. Results and Discussion

As a capping agent, taurine provided stabilization, and detailed characterization was done by using standard techniques for determining the physico-chemical properties of metal nanoparticles [28]. The size, shape, size distribution, functional groups, and surface composition were evaluated for the taurine-capped metal nanoparticles.

### 3.1. UV-VIS-NIR Measurements

**Figure 2:**
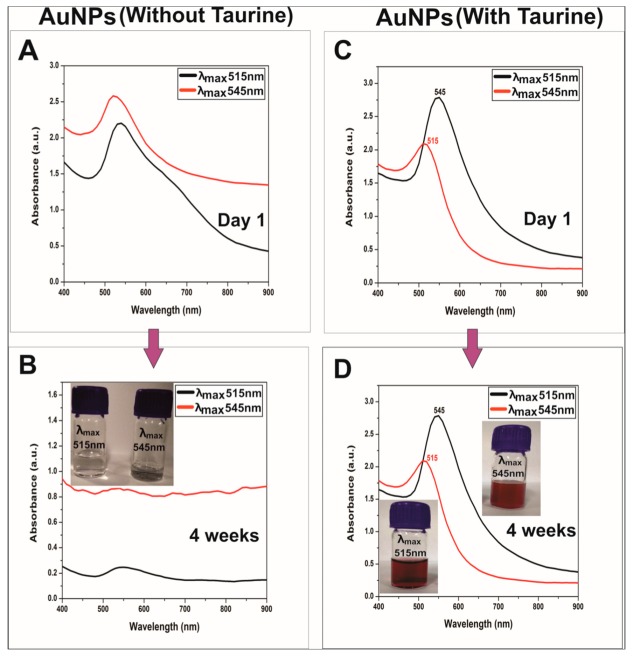
Optical spectroscopy and stability of gold nanospheres absorbing at 515 nm and 545 nm synthesized with/without taurine. Control samples (without taurine) showed aggregation within four weeks, as shown in (**B**). (**A**) show the formation of gold nanospheres on day one, but the shoulder peaks indicate a slight aggregation within the control samples. In contrast, the gold nanospheres capped with taurine had stable tunable plasmon bands from day one up to four weeks, as shown in (**C**,**D**). The inset images show the disappearance of color for the gold nanoparticle solutions without taurine, whereas the nanoparticles capped with taurine maintained stable colloidal gold nanoparticle solutions for up to four weeks.

Tunable sizes of the taurine-capped gold nanospheres were synthesized by using the wet-chemical reduction method. Figure 2 shows the absorbance bands of the gold that were nanoparticles synthesized with/without taurine. The different absorption bands (515 nm and 545 nm) were due to different sizes of the nanoparticles. Temperature played a major role in tuning the size of the nanoparticles that absorbed light in the visible wavelength of the electromagnetic spectrum. We also observed a color variation in both the colloidal solution that could have been attributed to their difference in surface plasmon resonance (SPR). This was due to the collective oscillations of the free electrons, a phenomenon that is referred to as localized surface plasmon resonance (LSPR) [2]. The nanoparticles that were synthesized with/without taurine had differences in their color and plasmon band stability. The taurine capped gold nanoparticles were stable for a month upon storage. The tunable absorbencies of the taurine-capped gold nanospheres were a result of temperature variation. The gold nanospheres that were synthesized at different temperatures resulted in tunable sizes with absorbencies at 515 nm and 545 nm. Figure 2A,B represents the control samples (without taurine) that were synthesized by using gold chloride as a metal salt and their subsequent reduction with sodium borohydride (strong reducing agent). The absorbance bands of the control samples had shoulder absorbance peaks approximately in the vicinity of 650 nm, thus indicating the presence of aggregates, as shown in Figure 2A. The color of the control samples disappeared within four weeks, as shown in Figure 2B. The absorbance bands of control samples disappeared within four weeks, thus indicating aggregation due to a lack of capping agent, as shown in Figure 2B. In contrast, the gold nanospheres capped with taurine showed absorbance bands that were stable for four weeks, as shown in Figure 2C,D. The taurine-capped AuNPs had tunable absorbance bands at 515 nm and 545 nm, as shown in Figure 2C, which depicts different sizes of gold nanospheres. The absorption bands were characteristic of the specific nanoparticle sizes and shapes. The taurine-capped gold nanosphere solutions showed stable colors, as shown in Figure 2D.

The difference in the nucleation and kinetic growth of the gold ions when reduced at certain temperatures led to different sizes of gold nanospheres. It is well-known that the kinetic energy of a molecule is a function of temperature, i.e., kinetic energy increases linearly with temperature, eventually leading to a higher nucleation rate. Therefore, the nucleation process at 100 °C is rapid due to the exhaustion of metal ions that convert into nanoparticle formations. In contrast, at lower temperatures, the rapid reduction of metal ions is slow and therefore leads to larger sized nanoparticles. The monodispersity and high yield of gold nanospheres is a result of maintaining ideal ratios between taurine and gold metal salt, along with controlled reaction parameters. The stoichiometric concentrations/volumes of the reagents used were critical in determining the size, shape and yield of the taurine-capped gold nanoparticles. The molar concentrations were critical because the absence of taurine led to the aggregation of nanoparticles along with color disappearance from nanoparticle solutions (referred as the control samples in Figure 2A,B). In addition, there were also concerns regarding the safety of sodium borohydride for biological applications when used as a strong reducing agent for nanoparticle synthesis [29]. However, using taurine as capping agent for metal nanoparticles provides an ease of synthesis and size tuning, along with excellent biocompatibility.

The gold nanoparticles without capping aggregated in different buffers when sodium borohydride was used for gold nanoparticle synthesis. In addition, the functionalization of metal nanoparticles with biomolecules is a limitation due to the absence of capping [30]. Similar reports on silver nanoparticle synthesis while using sodium borohydride have shown that it causes instability in nanoparticles and leads to aggregation via coalescence phenomena [31]. Gold nanoparticles that are synthesized only with NaBH_4_ require controlled synthetic conditions like stirring in the dark for one hour and further storage at 4 °C for stable nanoparticles [32]. Synthesizing nanoparticles with larger diameters leads to the formation of aggregates, and size tuning is a complex and laborious procedure. Therefore, using taurine as a capping agent for synthesis is a facile and rapid method for synthesizing stable and tunable sized gold nanospheres. The excess sodium borohydride can be washed via centrifugation to avoid aggregates and ROS formation. Taurine-capped AuNPs are reproducible and stable when stored at room temperature.

#### Stability of Gold Nanoparticles (with/without Taurine) in Phosphate Buffer Saline (PBS)

**Figure 3:**
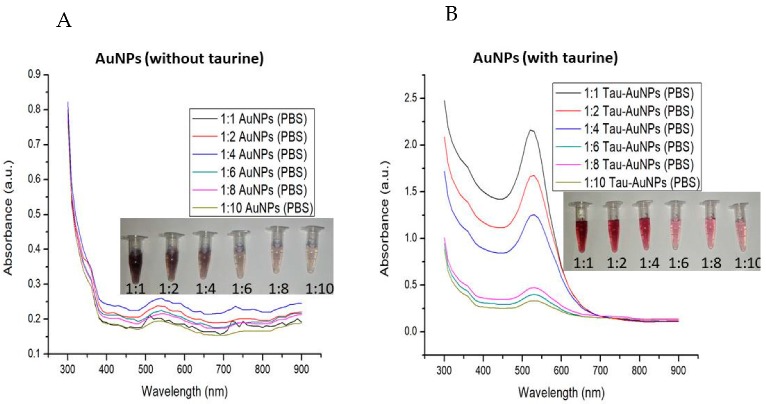
Stability of gold nanoparticles in phosphate buffer saline (PBS): (**A**) Gold nanoparticles synthesized in the absence of taurine showed plasmon peaks that were aggregated, and (**B**) gold nanoparticles synthesized in the presence of taurine showed stable plasmon bands when dispersed in PBS.

We evaluated the gold nanoparticles’ (with/without taurine) stability in PBS to determine the importance and differential behaviors between capped and uncapped nanoparticles. For biological applications, knowledge of the dispersity of the nanoparticles in PBS is critical to determine the monodispersity of nanoparticles during treatment of biological cells. Phosphate buffered saline is essential for the growth of cells and for maintaining homeostasis within cells. The nanoparticles synthesized by using taurine showed an excellent stability in PBS (pH 7.4) without any aggregation at different ratios, as shown in Figure 3B. The gold nanoparticles synthesized without taurine as capping agent underwent aggregation, as shown in Figure 3A [33].

### 3.2. ZetaSizer and NanoSight Characterization

**Figure 4:**
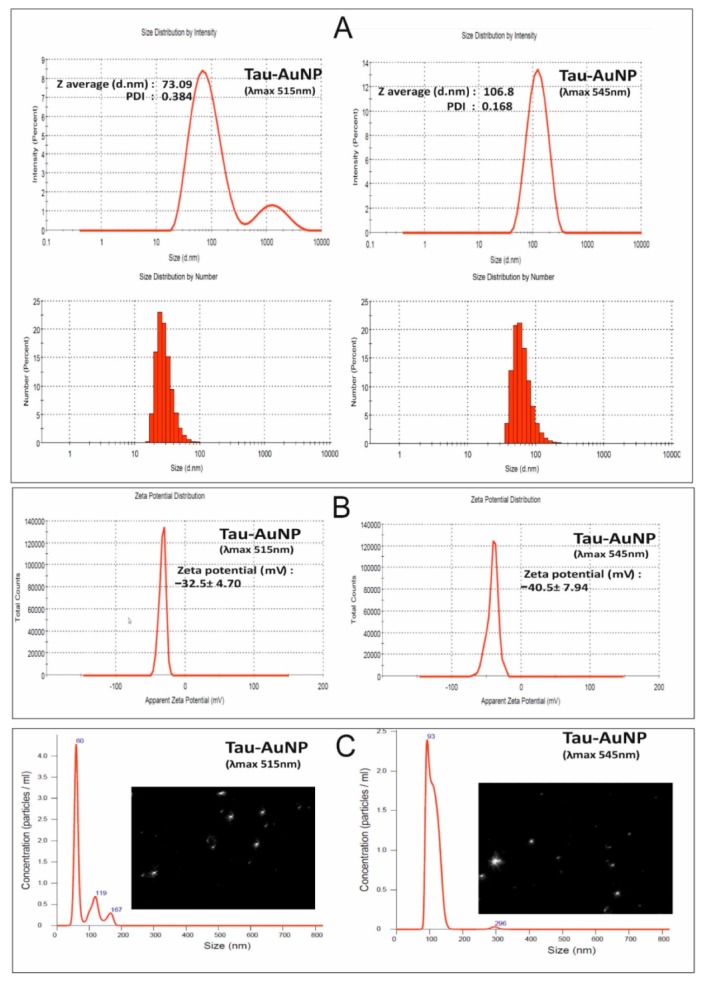
Dynamic Light Scattering DLS measurements showed the average size 73.9 and 106.8 nm with polydispersity index (PDI) values of 0.384 and 0.168, respectively, for the taurine-capped AuNP with λ_max_515 and λ_max_545 nm. The histograms show the size distribution of the taurine-capped metal gold nanoparticles (AuNPs), as shown in (**A**). Zeta potential measurements show a negative surface charge for the taurine-capped metal gold nanospheres at pH 3.30, as shown in (**B**). (**C**) Nanoparticle tracking analysis (NTA) measurements for taurine-capped gold nanoparticles determining their average size and Brownian motion (white spots depict nanoparticle motion).

The taurine-capped gold nanoparticles (λ_max_515 and λ_max_545 nm) were characterized by using a ZetaSizer and a NanoSight to measure their average hydrodynamic radius, polydispersity index (PDI), surface charge and concentration. The Dynamic Light Scattering (DLS) measurements showed that the average hydrodynamic radius and PDI for the nanoparticles absorbing at λ_max_515 and λ_max_545 nm were 73.09 nm (PDI: 0.38) and 106.8 nm (PDI: 0.16), respectively, as shown in Figure 4A. The inset images show histograms (size distribution) for the samples that indicate monodispersity. The difference in the average hydrodynamic radius of the synthesized gold nanospheres indicated that the nanoparticles had different size dimensions. The PDI of the taurine-capped gold nanoparticles showed good dispersity and the absence of aggregates in the nanoparticle solutions.

The zeta potential of nanoparticles represents the electrical double layer and determines their electrical properties between solid/liquid interfaces. The taurine-capped gold nanospheres (λ_max_515 nm and λ_max_545 nm) showed negative zeta potential values of −32.5 and −40.5 mV, respectively, and the pH was set at 3.30, as shown in Figure 4B. The surface charge is dependent on the nature of capping agent that is used for stabilizing the nanoparticles. Taurine has sulfonate groups that bear oxygen molecules that bind to the negatively charged gold metal surface. Therefore, gold nanospheres have an overall negative charge due to the capping with taurine. The surface charge of the synthesized gold nanospheres was measured with a ZetaSizer. The taurine-capped gold nanospheres (λ_max_515 nm and λ_max_545 nm) showed negative zeta potential values of −32.5 and −40.5 mV, respectively, at a pH of 3.30, as shown in Figure 4B. It is well known that nanoparticles with a zeta potential of (±) 30 mV in a colloidal solution show good stability because the similar surface charges repel each other, thus preventing aggregation [34]. In the present work, the higher negative zeta potential of −30 mV showed an excellent colloidal stability. The surface charge is dependent on the nature of capping agent that is used for stabilizing the nanoparticles. Therefore, the gold nanospheres had an overall negative charge due to the capping with taurine. A size characterization tool called nanoparticle tracking analysis (NTA) was introduced to acquire the size/concentration of the particles by determining their Brownian motion. The hydrodynamic radius was calculated by using diffusion coefficients based on the movements of individual particles in successive optical video images. We measured the concentration (particles/mL) of the nanoparticles with the aforementioned NTA. The Brownian motion of the nanoparticles was visualized (white spots), thus indicating that the presence of nanoparticles and the hydrodynamic sizes were 60 nm and 93 nm for nanoparticles absorbing at λ_max_515 nm and λ_max_545 nm, respectively, as shown in Figure 4C. However, the major drawback for this approach is the lack of determination for nanoparticle sizes of less than 30 nm [35]. Recently, a similar variability in the DLS and NTA measurements for nanoparticles were reported for polydisperse macromolecules, indicating that dissimilarities in the measurement of nanoparticles are dependent on several factors such as ionic strength, pH and dispersity [36,37]. Nanoparticles measured by using DLS/NTA generally take not only the nanoparticle but also the ionic and solvent layers in the solution phase into consideration. NTA and DLS measure the solution form of nanoparticles in Brownian motion to determine their hydrodynamic radius and concentration. The results are different in comparison to TEM because the nanoparticles are measured in their dried form [38].

### 3.3. Electron Microscopy Imaging

**Figure 5:**
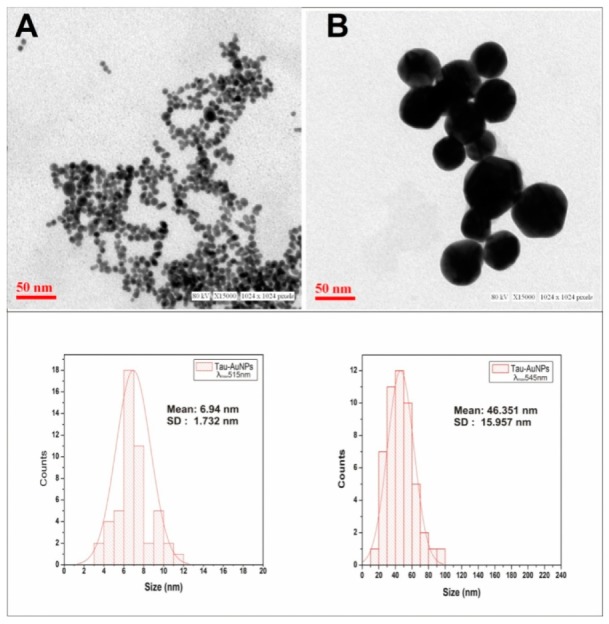
Electron microscopy for the visualization and size estimation of the taurine-capped gold nanospheres: Taurine-capped AuNPs with λ_max_515 nm had an average diameter of 6.9 nm, as shown in (**A**). Taurine-capped AuNPs with λ_max_545 nm had an average diameter of 46 nm, as shown in (**B**). Spherical shapes were observed for the samples, which were monodisperse. The inset Gaussian curve represents the mean size (50 nanoparticles per sample from TEM images were measured for statistics) of the gold nanospheres.

Figure 5 shows the electron microscopy (TEM) image of the taurine-capped gold nanospheres. The gold nanospheres had tunable absorbencies that indicated different sizes. TEM imaging evaluated the precise size of the formed nanospheres. The average mean size of the nanospheres was about 6.9 nm and 46.3 nm for the particles absorbing at 515 nm and 545 nm, respectively, as shown in Figure 5A,B. The histograms showed that the size distribution of nanoparticles had a good Gaussian distribution. Fifty nanoparticles per sample were measured for statistics to determine their average mean size. The nanospheres were monodisperse and free of aggregation. However, the size of the nanoparticles obtained via TEM had a considerable difference with that of the measurements obtained through DLS and NTA. This is because nanoparticles in colloidal solutions possess a hydrodynamic boundary layer and taurine, which is an organic compound when capped, aided in the prominent thickening of that layer. Moreover, for TEM imaging, the samples were not in colloidal form—rather, they were air dried on grids prior to observation. Thus, TEM imaging provided the actual size of the nanoparticles without consideration of the hydrodynamic layer.

### 3.4. Atomic Absorption Spectroscopy

The gold content of the synthesized, taurine-capped AuNPs of tunable sizes were quantified by using atomic absorption spectroscopy (AAS). The amount of gold determined was 84.34 and 41.61 µg/mL for nanoparticles that had plasmon bands at λ_max_515 nm and λ_max_545 nm respectively.

### 3.5. FTIR and Raman Analysis

**Figure 6:**
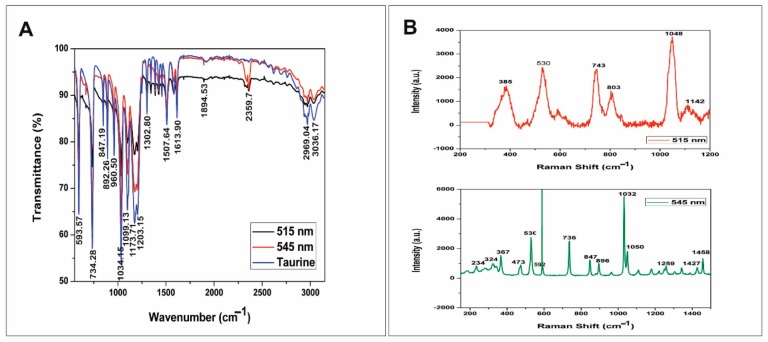
Spectra of the bulk compound taurine and the taurine-capped gold nanospheres are shown in (**A**). The characteristic bands represent the interactions between taurine and the gold nanospheres. (**B**) showed the Raman spectra of the λ_max_515 nm and λ_max_545 nm gold nanospheres capped with taurine, thus depicting their molecular vibrations.

Figure 6A shows the FTIR spectrum of the taurine-capped metal gold nanospheres and reveals the functional groups present on the nanoparticle surface. The taurine-capped gold nanoparticles exhibiting peaks at 3036 and 2969 cm^−1^ corresponded to the presence of OH group and the C–H stretching of alkanes, respectively [39]. The band at 1613 cm^−1^ showed the –C=N stretching of the amide group present in taurine [39]. In addition, greater absorption was observed on the broad spectral range of 1099–1302 cm^−1^ in the taurine-capped AuNPs, thus indicating interactions between the AuNPs [40]. The super imposing FTIR spectrum also suggested the successful capping of the gold nanoparticles with taurine with slight variation at the peak of 2359 cm^−1^.

The surface functionalization of the taurine-capped gold nanoparticles was validated by using Raman spectroscopy. Figure 6B shows prominent peaks in the vicinity of 1046, 742, 540, 530, and 353 cm^−1^. A very intense Raman band could be observed at 1046 cm^−1^ that was assigned to SO_3_ symmetric stretching vibration of taurine [41]. The band at 742 cm^−1^ was due to the C−S stretching region, which has also been used for the determination of the molecular conformation of sulfur-containing organic compounds [23]. The bands at 530 and 353 cm^−1^ corresponded to SO_3_ symmetric deformation and SO_3_ rocking, respectively, in the taurine molecules that capped the gold nanoparticle [42]. The results were similar to those reported in the literature for Raman bands, thus validating the interaction between taurine and noble metal nanoparticles [23]. The vibrational band obtained for the taurine-capped gold nanoparticles matched with the vibrational bands of bulk taurine, and this validates the interaction between taurine and the gold metal surface.

### 3.6. XRD and XPS Analysis

**Figure 7:**
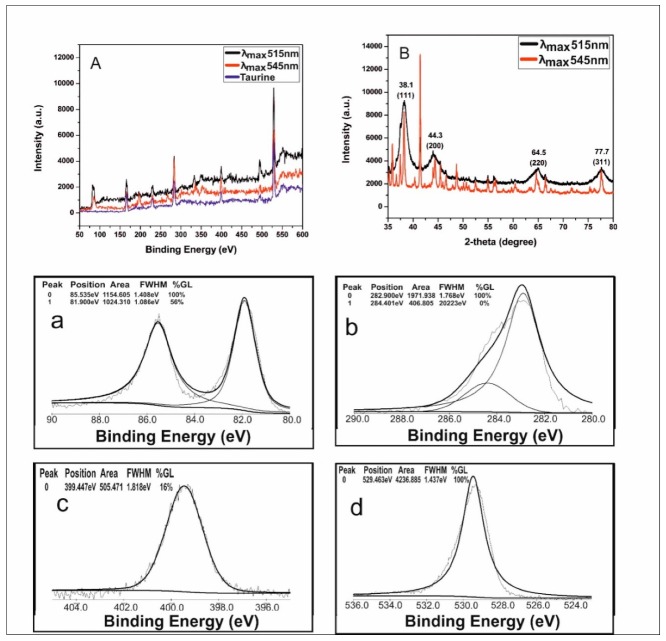
XPS spectra of the taurine-capped AuNPs (with λ_max_515 and λ_max_545 nm) and the bulk compound taurine as shown in (**A**). The inset graphs in (**a**–**d**) show the deconvolution of the Au4f, C1s, N1s, and O1s spectra, respectively. (**B**) XRD pattern of the taurine-capped gold nanospheres index at (111), (200), (220) and (311), depicting their crystalline nature for gold metal.

To evaluate the crystalline structure of the synthesized, taurine-capped metal gold nanoparticles, we performed X-ray diffraction (XRD) on the powdered samples with PANalytical XRD (IIT Kanpur), as shown in Figure 7B. The scan was taken for the 2θ range of 35–80 degrees. The tunable sizes of the taurine-capped gold nanospheres (λ_max_515 nm and λ_max_545 nm) were evaluated for the gold crystal structure. The diffractogram showed the peaks of 2θ values at 38.1, 44.3, 64.5 and 77.7 with Miller indices of (111), (200), (220) and (311), respectively, for the obtained peaks, as shown in Figure 7B. The analysis showed that the metal gold nanoparticles had face centered cubic (fcc) lattices. The results are similar to the standard powder diffraction card of JCPDS, (gold file No. JCPDS 04-0784), as reported elsewhere [40]. The intense peak at 2θ = 38.1 showed preferential growth in the {111} direction. The selective growth in {111} direction was a result of the molecular-sized crystals formed from repeated units of atoms at a given distance and time intervals. The mean crystalline size was calculated with the Debye–Scherrer formula:

D = 0.89λ/βcosθ,

where D is the mean crystalline size, λ is the wavelength of the XRD, β is the full width at half maximum (FWHM), and θ is the diffraction angle. The structural parameters of the synthesized metal gold nanoparticles are shown in Table 1 and Table 2, respectively.

### 3.7. XPS Analysis

To understand the role of taurine capping to metal gold nanoparticle surfaces, we evaluated their binding affinity via X-ray photon spectroscopy measurements. The percentage composition of the elements present in the taurine-capped metal gold nanoparticles is shown in Figure 7A. The surface chemical state and surface composition of the taurine-capped AuNPs were analyzed by using the XPS technique. The XPS spectra (Figure 7A) showed an intense gold signal, confirming the presence of pure gold without any impurities. The peaks attributed to C, N, and O showed the presence of the amino acid taurine, which was capped over the surface of AuNPs. Figure 7A,a shows the enlarged view of the Au-4f core-level spectrum, which presented two peaks (at 81 and 86 eV). These two peaks corresponded to the Au (4f_5/2_) and Au (4f_7/2_) transitions, respectively. The obtained peaks were assigned to the spin-orbit splitting component of the zero valent gold Au (4f_7/2_), while the binding energies of 81 and 86 eV for the Au (4f_7/2_) corresponded to Au^+^ and Au^3+^, respectively, as reported previously [43]. The enlargement of the C1s, N1s and O1s spectra shown in Figure 7 b–d correspond to binding energies of 283, 399 and 528.5 eV respectively, confirming the presence of an organic compound, i.e., taurine on the synthesized AuNPs. The binding of taurine via the oxygen atoms of the –S–OH group was validated for their strong interaction. Our results were similar to those previously reported in the literature [44] where hydroxyl group have been found to have stronger affinity to gold surfaces. The percentage of surface composition revealed the presence of 76% Au–O in the taurine-capped gold nanoparticles. Ours is the first report on the interaction between gold nanoparticle surfaces and taurine.

### 3.8. Cell Viability Assay

**Figure 8:**
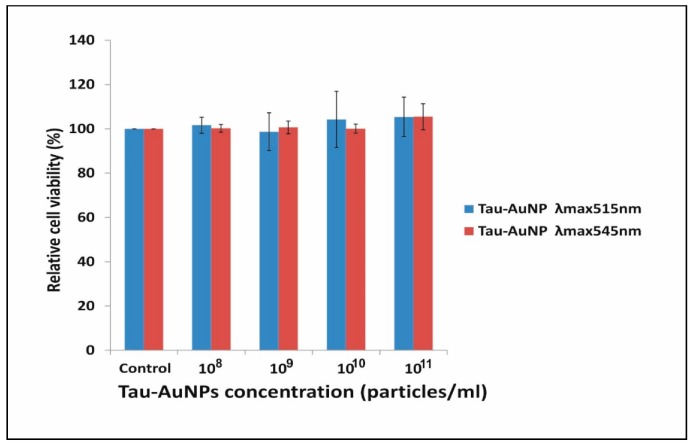
In vitro cell viability assessment of taurine-capped gold nanospheres with absorbance λ_max_515 nm and λ_max_545 nm by using an MTT assay. Liver carcinoma cells (HepG2) were exposed to different concentrations (particles/mL) of gold nanospheres with different sizes.

There are many inconclusive data in the literature regarding whether gold nanoparticles are toxic or non-toxic. The toxicity of nanoparticles is also influenced by the size and shape of nanoparticles, along with their surface chemistry. Some nanoparticles have shown size-dependent toxicity, whereas others have shown surface chemistry-dependent toxicity [45]. Therefore, predicting toxicity is difficult because similar sizes of nanoparticles results in differential toxicity on the same cell line. Surface chemistry plays a critical role because positively charged nanoparticles are easily uptaken by cells in comparison to negatively charged nanoparticles [46]. Unlike anionic capping (citrate, tannic acid, polystyrene sulfonate and polyvinylpyrrolidine), taurine derivatives were also reported for its excellent biocompatibility and used as additives in dental implants [47]. In the present study, we assessed the safety of taurine capped gold nanoparticles on liver carcinoma cells as liver is the major organ for accumulation of nanoparticles causing hepato-toxicity [46]. Two different sizes (6.9 nm and 46 nm) of taurine-capped AuNPs were exposed to the liver carcinoma cell line in concentration range of 10^8^ and 10^11^ particles/mL, as shown in Figure 8. Both sizes of the gold nanospheres were non-toxic irrespective of their size, thus indicating that taurine capping is biocompatible. Taurine can be an effective capping agent for gold nanoparticles, as their size has no effect on cell death. Cationic-capped metal gold nanoparticles also elicited toxic responses towards liver cancer cells due to their positive charge that led to the uptake of nanoparticles [46]. In our case, we used a non-protein amino acid that was naturally synthesized within the body and has tremendous potential for therapeutic applications in various disease conditions. We would like to emphasize that widely reported, non-toxic molecules like citrate were actually found to be toxic and mostly genotoxic at low concentrations when exposed to liver cells [16]. Therefore, the nature of the nanoparticles in respect to capping agents may differ and have different responses towards biological cells. However, to increase the bioavailability of gold nanoparticles, taurine, as a capping agent, can behave as a nano-carrier in order to release itself and provide specific targeting capabilities. Therefore, taurine can be an effective capping agent for synthesizing non-cytotoxic metal nanoparticles and has the potential to be explored for biomedical and clinical applications.

## 4. Conclusions

In conclusion, we report, for the first time, the use of taurine as a novel capping agent for gold nanospheres by using a facile one-step/one-pot wet-chemical method. The taurine-capped gold nanospheres were tunable in the visible wavelength at 515 nm and 545 nm and had average diameters of 6.9 nm and 46 nm, respectively. The critical parameter was temperature, which played a key role in tuning the size of gold nanospheres. Their size, shape and surface chemistry were evaluated by using optical spectroscopy, DLS, zeta potential, and electron microscopy. The interaction between taurine and gold metal surfaces was studied with the FTIR and Raman techniques. The XRD technique revealed the face centered cubic (fcc) crystal structure of the taurine-capped gold nanoparticles. The binding energy and elemental composition of the taurine and gold metal surfaces was confirmed via XPS, showing a 76% binding efficiency between oxygen and gold. Preliminary cytotoxicity studies (MTT assay) showed that the taurine stabilized gold nanospheres were non-toxic to liver carcinoma cell lines (HepG2) and were size-independent. We propose taurine as a novel non-cytotoxic capping agent that can be an alternative to existing toxic capping agents for metal gold nanoparticles. These taurine-capped metal gold nanoparticles have the potential for plausible applications in the area of nanomedicine and drug-delivery applications.

## Figures and Tables

**Table 1:** Structure parameters of the taurine-capped AuNPs with λ_max_515 nm.

No.	B Obs. (°2Th)	B Std. (°2Th)	Peak Pos. (°2Th)	B Structure (°2Th)	Crystallite Size (Å)	Crystallite Size (nm)
**1**	0.076	0	38.4	0.076	1107	110.7
**2**	0.102	0	43.9	0.102	840	84
**3**	0.307	0	65.05	0.307	307	30.7
**4**	0.187	0	77.6	0.187	545	54.5

**Table 2:** Structure parameters of the taurine-capped AuNPs with λ_max_545 nm.

No.	B Obs. (°2Th)	B Std. (°2Th)	Peak Pos. (°2Th)	B Structure (°2Th)	Crystallite Size (Å)	Crystallite Size (nm)
**1**	0.115	0	38.2	0.115	731	73
**2**	0.109	0	44.4	0.109	787	78
**3**	0.093	0	64.58	0.093	1010	101
**4**	0.109	0	77.61	0.109	935	93

## References

[1] Guerrero-Martínez A., Alonso-Gómez J.L., Auguié B., Cid M.M., Liz-Marzán L.M. (2011). From individual to collective chirality in metal nanoparticles. Nano Today.

[2] Burrows N.D., Lin W., Hinman J.G., Dennison J.M., Vartanian A.M., Abadeer N.S., Murphy C.J. (2016). Surface chemistry of gold nanorods. Langmuir.

[3] Qin Z., Wang Y., Randrianalisoa J., Raeesi V., Chan W.C., Lipiński W., Bischof J.C. (2016). Quantitative comparison of photothermal heat generation between gold nanospheres and nanorods. Sci. Rep..

[4] Scholl J.A., Koh A.L., Dionne J.A. (2012). Quantum plasmon resonances of individual metallic nanoparticles. Nature.

[5] Prodan E., Nordlander P., Halas N.J. (2003). Electronic structure and optical properties of gold nanoshells. Nano Lett..

[6] Huang F., Baumberg J.J. (2010). Actively tuned plasmons on elastomerically driven Au nanoparticle dimers. Nano Lett..

[7] Chithrani B.D., Ghazani A.A., Chan W.C. (2006). Determining the size and shape dependence of gold nanoparticle uptake into mammalian cells. Nano Lett..

[8] Soliman M.G., Pelaz B., Parak W.J., Del Pino P. (2015). Phase transfer and polymer coating methods toward improving the stability of metallic nanoparticles for biological applications. Chem. Mater..

[9] Khlebtsov N., Dykman L. (2011). Biodistribution and toxicity of engineered gold nanoparticles: A review of in vitro and in vivo studies. Chem. Soc. Rev..

[10] Raveendran P., Fu J., Wallen S.L. (2003). Completely “green” synthesis and stabilization of metal nanoparticles. J. Am. Chem. Soc..

[11] Kim D., Park S., Lee J.H., Jeong Y.Y., Jon S. (2007). Antibiofouling polymer-coated gold nanoparticles as a contrast agent for in vivo X-ray computed tomography imaging. J. Am. Chem. Soc..

[12] Duan H., Wang D., Li Y. (2015). Green chemistry for nanoparticle synthesis. Chem. Soc. Rev..

[13] Iravani S. (2011). Green synthesis of metal nanoparticles using plants. Green Chem..

[14] Sau T.K., Pal A., Jana N.R., Wang Z.L., Pal T. (2001). Size controlled synthesis of gold nanoparticles using photochemically prepared seed particles. J. Nanopart. Res..

[15] Wang A., Ng H.P., Xu Y., Li Y., Zheng Y., Yu J., Fu L. (2014). Gold nanoparticles: Synthesis, stability test, and application for the rice growth. J. Nanomater..

[16] Fraga S., Faria H., Soares M.E., Duarte J.A., Soares L., Pereira E., Costa-Pereira C., Teixeira J.P., de Lourdes Bastos M., Carmo H. (2013). Influence of the surface coating on the cytotoxicity, genotoxicity and uptake of gold nanoparticles in human HepG2 cells. J. Appl. Toxicol..

[17] Zeng Q., Shao D., Ji W., Li J., Chen L., Song J. (2014). The nanotoxicity investigation of optical nanoparticles to cultured cells in vitro. Toxicol. Rep..

[18] Liu P., Ge X., Ding H., Jiang H., Christensen B.M., Li J. (2012). Role of glutamate decarboxylase-like protein 1 (GADL1) in taurine biosynthesis. J. Biol. Chem..

[19] Nakajima Y., Osuka K., Seki Y., Gupta R.C., Hara M., Takayasu M., Wakabayashi T. (2010). Taurine reduces inflammatory responses after spinal cord injury. J. Neurotrauma.

[20] Schaffer S., Kim H.W. (2018). Effects and mechanisms of taurine as a therapeutic agent. Biomol. Ther..

[21] Jakaria M., Azam S., Haque M.E., Jo S.H., Uddin M.S., Kim I.S., Choi D.K. (2019). Taurine and its analogs in neurological disorders: Focus on therapeutic potential and molecular mechanisms. Redox Biol..

[22] Yeh Y.H., Lee Y.T., Hsieh H.S., Hwang D.F. (2009). Effect of taurine on toxicity of aluminum in rats. e-SPEN Eur. E-J. Clin. Nutr. Metab..

[23] Maiti N., Thomas S., Debnath A., Kapoor S. (2016). Raman and XPS study on the interaction of taurine with silver nanoparticles. RSC Adv..

[24] Huxtable R.J. (1992). Physiological actions of taurine. Physiol. Rev..

[25] O’Donnell C.P., Allott K.A., Murphy B.P., Yuen H.P., Proffitt T.M., Papas A., Moral J., Pham T., O’Regan M.K., Phassouliotis C. (2016). Adjunctive Taurine in First-Episode Psychosis: A Phase 2, Double-Blind, Randomized, Placebo-Controlled Study. J. Clin. Psychiatry.

[26] Mosmann T. (1983). Rapid colorimetric assay for cellular growth and survival: Application to proliferation and cytotoxicity assays. J. Immunol. Methods.

[27] Rayavarapu R.G., Petersen W., Hartsuiker L., Chin P., Janssen H., van Leeuwen F.W., Otto C., Manohar S., van Leeuwen T.G. (2010). In vitro toxicity studies of polymer-coated gold nanorods. Nanotechnology.

[28] Mourdikoudis S., Pallares R.M., Thanh N.T. (2018). Characterization techniques for nanoparticles: Comparison and complementarity upon studying nanoparticle properties. Nanoscale.

[29] Datta L.P., Chatterjee A., Acharya K., De P., Das M. (2017). Enzyme responsive nucleotide functionalized silver nanoparticles with effective antimicrobial and anticancer activity. New J. Chem..

[30] Thompson A.B., Calhoun A.K., Smagghe B.J., Stevens M.D., Wotkowicz M.T., Hatziioannou V.M., Bamdad C. (2011). A gold nanoparticle platform for protein–protein interactions and drug discovery. ACS Appl. Mater. Interfaces.

[31] Bhattarai N., Khanal S., Pudasaini P.R., Pahl S., Romero-Urbina D. (2011). Citrate stabilized silver nanoparticles: Study of crystallography and surface properties. Int. J. Nanotechnol. Mol. Comput..

[32] Chen W., Deng H.H., Hong L., Wu Z.Q., Wang S., Liu A.L., Lin X.H., Xia X.H. (2012). Bare gold nanoparticles as facile and sensitive colorimetric probe for melamine detection. Analyst.

[33] Du S., Kendall K., Toloueinia P., Mehrabadi Y., Gupta G., Newton J. (2012). Aggregation and adhesion of gold nanoparticles in phosphate buffered saline. J. Nanopart. Res..

[34] Tantra R., Schulze P., Quincey P. (2010). Effect of nanoparticle concentration on zeta-potential measurement results and reproducibility. Particuology.

[35] De Temmerman P.J., Verleysen E., Lammertyn J., Mast J. (2014). Size measurement uncertainties of near-monodisperse, near-spherical nanoparticles using transmission electron microscopy and particle-tracking analysis. J. Nanopart. Res..

[36] Filipe V., Hawe A., Jiskoot W. (2010). Critical evaluation of Nanoparticle Tracking Analysis (NTA) by NanoSight for the measurement of nanoparticles and protein aggregates. Pharm. Res..

[37] Kim A., Ng W.B., Bernt W., Cho N.J. (2019). Validation of size estimation of Nanoparticle tracking Analysis on polydisperse Macromolecule Assembly. Sci. Rep..

[38] Eaton P., Quaresma P., Soares C., Neves C., de Almeida M.P., Pereira E., West P. (2017). A direct comparison of experimental methods to measure dimensions of synthetic nanoparticles. Ultramicroscopy.

[39] Huang P., Li J., Liu X., Wu F. (2016). Colorimetric determination of aluminum (III) based on the aggregation of Schiff base-functionalized gold nanoparticles. Microchim. Acta.

[40] Dubey S.P., Lahtinen M., Sillanpää M. (2010). Tansy fruit mediated greener synthesis of silver and gold nanoparticles. Process Biochem..

[41] Anderson J.M., Johnson R.L., Friedel K., Trunschke A., Schlögl R., Schmidt-Rohr K., Shanks B.H. (2014). Hydrothermally Stable Heterogeneous Catalysts for Biorenewable-Derived Molecule Conversions to Chemicals. Ph.D. Thesis.

[42] Moreira R.L., Lobo R.P., Dias A. (2018). Infrared dispersion analysis and Raman scattering spectra of taurine single crystals. Spectrochim. Part A Mol. Biomol. Spectrosc..

[43] Jaramillo T.F., Baeck S.H., Cuenya B.R., McFarland E.W. (2003). Catalytic activity of supported Au nanoparticles deposited from block copolymer micelles. J. Am. Chem. Soc..

[44] Lin Z., Wu J., Xue R., Yang Y. (2005). Spectroscopic characterization of Au^3+^ biosorption by waste biomass of *Saccharomyces cerevisiae*. Spectrochim. Part A Mol. Biomol. Spectrosc..

[45] Iswarya V., Manivannan J., De A., Paul S., Roy R., Johnson J.B., Mukherjee A. (2016). Surface capping and size-dependent toxicity of gold nanoparticles on different trophic levels. Environ. Sci. Pollut. Res..

[46] Agrahari K., Rayavarapu R.G. (2019). Chloride ions assisted synthesis of tunable gold nanorods: Seedless synthesis, characterization and in vitro toxicity studies. Vacuum.

[47] John G., Schwarz F., Becker J. (2015). Taurolidine as an effective and biocompatible additive for plaque-removing techniques on implant surfaces. Clin. Oral Investig..

